# Microscale Metal Additive Manufacturing by Solid‐State Impact Bonding of Shaped Thin Films

**DOI:** 10.1002/smll.202503014

**Published:** 2025-07-14

**Authors:** Alain Reiser, Christopher A. Schuh

**Affiliations:** ^1^ Department of Materials Science and Engineering Massachusetts Institute of Technology Cambridge MA 02139 USA; ^2^ Department of Materials Science and Engineering KTH Royal Institute of Technology Stockholm 114 28 Sweden; ^3^ Department of Materials ETH Zürich Zürich 8093 Switzerland; ^4^ Department of Materials Science and Engineering Northwestern University Evanston IL 60208 USA

**Keywords:** 3D printing, additive manufacturing, impact bonding, laser‐induced forward transfer, metals, microfabrication, particle impact testing, thin films

## Abstract

The deposition of device‐grade inorganic materials is one key challenge toward the implementation of additive manufacturing (AM) in microfabrication, and to that end, a broad range of physico‐chemical principles has been explored for 3D fabrication with micro‐ and nanoscale resolution. Yet, for metals, a process that achieves material quality rivalling that of established thin‐film deposition methods, and at the same time, has the potential to combine high throughput production with a broad palette of processable materials, is still lacking. Here, the kinetic, solid‐state bonding of metal thin films for the additive assembly of high‐purity, high‐density metals with micrometer‐scale precision is introduced. Indirect laser ablation accelerates micrometer‐thick gold films to hundreds of meters per second without their heating or ablation. Their subsequent impact on the substrate above a critical velocity forms a permanent, metallic bond in the solid state. Stacked layers are of high density (>99%). By defining thin‐film layers with established lithographic methods prior to launch, a variable feature size (2–50 µm), arbitrary shape of bonded layers, and parallel transfer of up to 36 independent film units in a single shot, is demonstrated. Thus, the solid‐state kinetic bonding principle as a viable and potentially versatile route for micro‐scale AM of metals is established.

## Graphical abstract

The deposition of device‐grade metals is one key challenge toward additive micromanufacturing. The impact‐induced solid‐state bonding of metal thin films for the additive assembly of high‐purity, high‐density metals with micrometer‐scale precision, is introduced. Demonstrating a variable feature size and arbitrary shapes of transferred layers, the solid‐state kinetic bonding principle as a viable and potentially versatile route for micro‐scale additive manufacturing of metals is established.

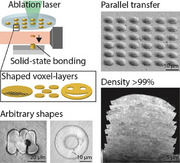

## 1 Introduction

At the micro‐ and nanoscale, the replacement of well‐established planar materials structures with 3D geometries enables exciting growth in fields ranging from biomedicine to metamaterials.^[^ ^1^ ^]^ Importantly, progress in microscale 3D engineering follows from the ability to realize the desired 3D structures from relevant materials. Among all strategies for 3D micro‐ and nanofabrication, the concept of additive manufacturing (AM)^[^ ^2^, ^3^, ^4^ ^]^ offers the highest complexity and most rigorous control of geometry (compared for example to stress‐controlled deformation or self‐assembly),^[^ ^1^ ^]^ and has established its potential in various fields, from lattice^[^ ^5^, ^6^ ^]^ and metamaterials^[^ ^7^, ^8^, ^9^, ^10^ ^]^ to 3D probes,^[^ ^11^, ^12^, ^13^, ^14^ ^]^ interconnects,^[^ ^15^, ^16^, ^17^ ^]^ and electrochemical storage devices.^[^ ^18^, ^19^, ^20^ ^]^

In metals, a broad range of physico‐chemical principles has been explored for additive deposition with micro‐ and nanoscale resolution (feature size <10 µm). Major technology families for the direct and indirect fabrication of metal structures include the transfer and sintering of colloids (direct ink writing (DIW);^[^ ^15^, ^21^ ^]^ electrohydrodynamic printing;^[^ ^22^, ^23^ ^]^ laser‐induced forward transfer (LIFT);^[^ ^24^, ^25^ ^]^ or aerosol printing^[^ ^26^, ^27^ ^]^), two‐photon‐lithography (TPL) of metal‐loaded resists^[^ ^28^, ^29^, ^30^ ^]^ or organic templates that are then metallized post deposition,^[^ ^5^, ^29^, ^31^, ^32^ ^]^ two‐photon reduction of metal salts,^[^ ^33^, ^34^ ^]^ electrochemical methods,^[^ ^16^, ^35^, ^36^, ^37^ ^]^ and focused electron‐ and ion‐induced deposition akin to chemical vapour deposition processes.^[^ ^38^ ^]^ Of these, the printing of colloids and TPL methods are most widespread, with TPL outperforming competing technology in throughput by orders of magnitude (when normalized by printing resolution^[^ ^2^, ^39^ ^]^). But as an important limitation, these methods require post‐print thermal conversion and/or consolidation to render cohesive metal structures. In all cases, high‐temperature post processing poses multiple materials challenges: incomplete and inhomogeneous sintering can cause significant residual porosity;^[^ ^40^ ^]^ excessive linear shrinkage, especially in TPL routes that rely on thermal oxidation and reduction steps (on the order of 60–80%^[^ ^5^, ^29^ ^]^), severely complicates the damage‐free and high‐fidelity consolidation of structures that are attached to noncompliant substrates; and sintering temperatures of 200–400 °C,^[^ ^40^ ^]^ or reduction, calcination and pyrolysis temperatures of 600–1000 °C,^[^ ^5^, ^29^ ^]^ are prohibitive for seamless integration with CMOS manufacturing processes.

Electrochemical^[^ ^36^ ^]^ and photoelectrochemical^[^ ^34^ ^]^ microscale AM (µAM) methods that directly synthesize dense and high‐purity metals circumvent the challenges of post‐deposition heat treatment, and generally offer materials performance that is competitive with thin‐film materials deposited by established microfabrication technology.^[^ ^16^, ^40^, ^41^ ^]^ Room‐temperature, one‐step synthesis further offers the advantage of local modulation of composition^[^ ^35^ ^]^ or microstructure,^[^ ^42^, ^43^ ^]^ while the need for conductive substrates can be navigated by thin seed layers or the use of electron‐beams as electron sources.^[^ ^44^ ^]^ Yet, slow growth kinetics limit deposition rates to 1–3 orders of magnitude below that of even the slowest ink‐based methods.^[^ ^2^, ^36^ ^]^ As a consequence of these challenges, metal µAM processes and their materials cannot yet satisfy the standards of state‐of‐the‐art microfabrication.^[^ ^40^ ^]^ Major handicaps toward the integration of µAM in advanced microfabrication are scalability and limited throughput, but also a lack of a large variety of device‐grade inorganic materials of highest quality that are deposited under conditions that are intrinsically compatible with established substrates and processes.

We address this overarching challenge of 3D microprinting high‐quality inorganic materials at high rates by advancing a new strategy: room‐temperature, kinetic bonding of laser‐accelerated metal‐thin films, or voxels, in the solid state. At macroscopic length scales, solid‐state bonding upon high‐velocity impact is a proven principle for the formation of high‐performance metal coatings and joints, routinely used at industrial scale in processes such as impact welding or cold spray.^[^ ^45^, ^46^ ^]^ In cold spray, microparticles form metallic bonds upon severe plastic co‐deformation during impact at hundreds of meters per second. Kinetic bonding is thus a principle that fuses and consolidates microparticles, but is not reliant on thermal input or diffusive processes (as sintering‐based consolidation is). As such, it has potential to circumvent the thermal post‐processing that is often problematic at small scales. Yet, the poor spatial resolution of gas‐accelerated particle sprays—millimeters for standard cold spray and ≈100 µm for miniature nozzles^[^ ^47^ ^]^—prevents direct adaption of the cold spray principle in microscale printing. Thus, we look for a more precise form of high‐velocity particle transfer.

We have previously used laser‐acceleration of single metal particles for the serial impact‐bonding and stacking of metal particles with a precision on the order of 10 µm.^[^ ^48^ ^]^ However, that process, based on laser‐induced particle impact testing (LIPIT), is slow and still of inferior precision compared to established µAM. In a crucial step toward precise microscale printing, we herein turn toward the transfer of *thin films* instead of particles. In general, the acceleration of thin films by laser ablation is a broadly useful concept explored for materials testing^[^ ^49^ ^]^ as well as patterning. In the latter domain, LIFT of mainly nanoparticle ink films^[^ ^25^, ^50^ ^]^ and metal melts^[^ ^51^, ^52^ ^]^ has been a cornerstone of micro‐printing research for decades, owing to its microscale resolution, extreme versatility in terms of materials, and comparably high throughput achieved by congruent transfer of shaped layers.^[^ ^25^, ^50^, ^53^ ^]^

From these prior works follows an opportunity to combine the precise laser‐transfer of films (as in LIFT) with the kinetic bonding of metal films (as in LIPIT) to advance a new mode of µAM. This approach benefits from the ease of laser transfer but overcomes the materials limitations, mainly significant porosity,^[^ ^40^ ^]^ faced by ink^[^ ^25^, ^50^ ^]^ and liquid‐droplet^[^ ^51^, ^52^ ^]^ LIFT by exploiting bonding and densification of metals in the solid state. Here, the major obstacle toward 3D fabrication is an unambiguous demonstration of the solid‐state impact‐bonding principle for laser‐transferred metal thin films, allowing the buildup of multiple thick metal layers; the closest prior efforts involved only single layers, very thin layers, and generally without metallic bonding.^[^ ^53^, ^54^, ^55^, ^56^, ^57^ ^]^ We directly address this open challenge here, and demonstrate ballistic transfer and solid‐state bonding of micrometer‐thick, solid metal microvoxels shaped by lithography, and their layering with sub‐micrometer precision.

## 2 Results and Discussion

### 2.1 Additive Micromanufacturing by Laser‐Induced Forward Transfer of Solid Gold Thin‐Film Voxel Layers

**Figure 1:**
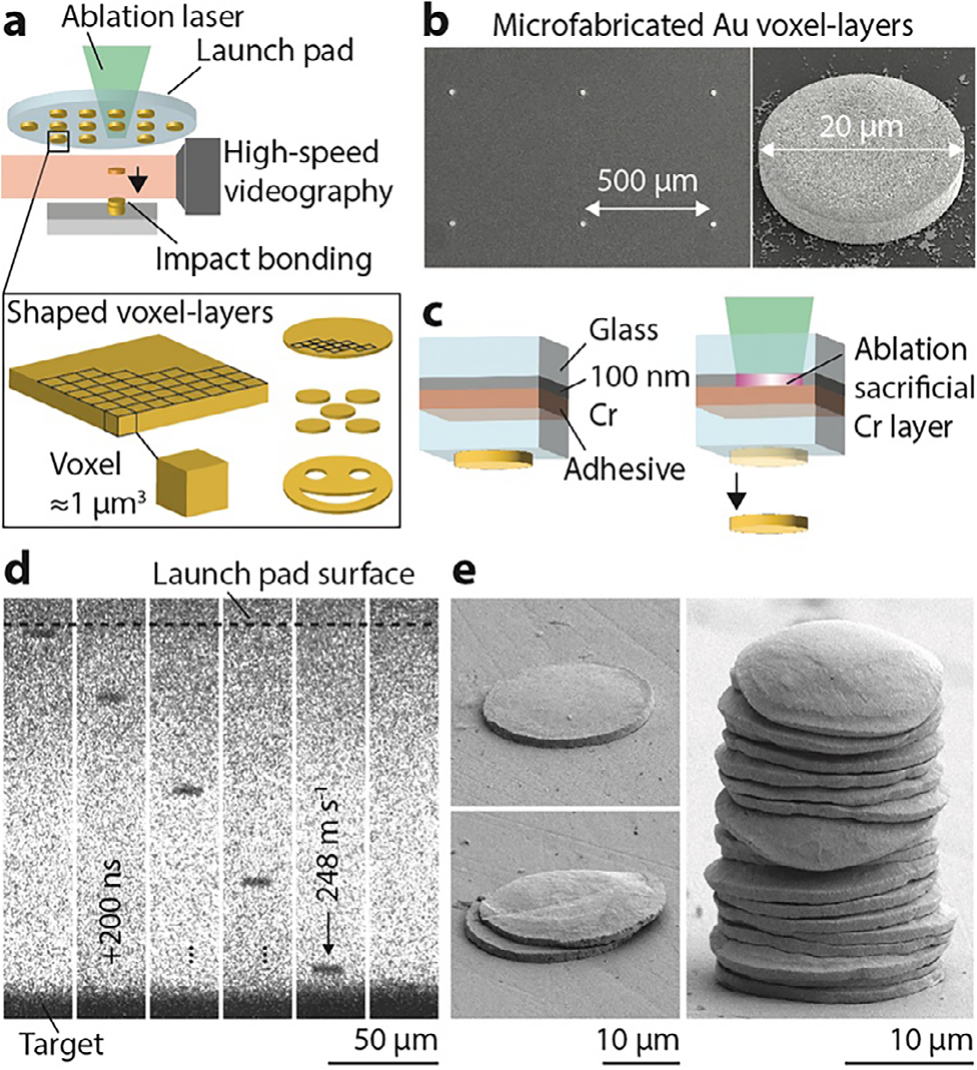
Solid‐state impact manufacturing of stacks of voxel layers. a) A solid gold thin‐film voxel layer is laser‐accelerated from a host substrate (launch pad) toward a target where it bonds upon impact. The flight and impact of layers are observed and quantified by synchronized, high‐speed videography. Arbitrary shapes and patterns of voxel layers may be defined on the launch pad by established microfabrication processes. b) SE‐SEM micrographs of an array of circular gold layers patterned by templated electroplating directly onto the launch pad. c) Launch of layers is induced by laser‐ablation of a sacrificial metal film buried within the launch pad architecture. This indirect, laser‐induced acceleration crucially guarantees the transfer of thin films with well‐defined thickness, shape and microstructure not affected by ablation. d) Optical image sequence (interframe time: 200 ns) of a gold thin film launched toward and bonded to a gold target at 248 m s^−1^. e) SE‐SEM micrographs of gold layers bonded to a gold target. Sequential transfer enables stacking of layers into 3D deposits (sample tilt: 70°, no tilt correction).

The deposition of 3D geometries is facilitated by the solid‐state bonding and stacking of gold thin‐films upon impact at velocities ≥200 m s^−1^ (**Figure** **1**a). To that end, gold films 0.5–6 µm in thickness and 2–50 µm in diameter are propelled from a “launch pad” by laser ablation of a sacrificial metal layer. High‐speed videography synchronized with the launch pulse enables direct observation of the flight and collision of the films with a gold target positioned 50–200 µm below the launch pad. Although not strictly necessary for micro‐AM, such high‐rate microscopy enables measurement of impact velocity, and this optical feedback could in principle be used for process quality monitoring in future iterations. The laser‐induced launch of high‐velocity microparticles and their imaging with nanosecond and micrometer spatio‐temporal resolution has been widely explored by laser‐induced particle impact testing (LIPIT),^[^ ^58^, ^59^ ^]^ and in fact we employ a LIPIT setup in combination with a custom launch pad architecture^[^ ^60^ ^]^ tailored to launch shaped thin films for these experiments. Metal films of defined shape were fabricated by electrodeposition into resist templates produced by lithography directly on the launch pad (Figure 1b). As schematically shown in (a), microfabrication permits the creation of well‐defined shapes or arrays of shapes that can be launched and bonded congruently. As will be discussed later in detail, these shapes may be the individual layers of the fabricated 3D geometry—layers that are composed of voxels whose size is defined by the pixel size of the lithography process multiplied by the film thickness (resulting in a voxel size on the order of 1 µm^3^). We thus term these films “voxel‐layers”. Their launch is achieved by ablation of a sacrificial metal layer buried inside the launch pad^[^ ^60^ ^]^ (Figure 1c), which offers multiple improvements over the previously described LIFT launch of solid films by direct ablation and “punch out” of nm‐thin donor metal films. First, thick films (up to 6 µm, instead of ≤ 400 nm for LIFT^[^ ^53^, ^54^, ^55^, ^56^, ^57^ ^]^) can be transferred. Second, the launch of films and their impact are highly parallel when properly aligned, and no deformation of the film in flight is observed (Figure 1d). Finally, melting, ablation or even heating of the transferred material is avoided altogether (additional layers in the launch pad can completely shield the voxel layer from irradiation with laser light if needed^[^ ^60^ ^]^). The latter is evidenced by the flat top surface and precisely defined edges of the transferred layers (Figure 1e). Thanks to the transfer in the solid state, the satellite splatters observed in liquid‐state LIFT are avoided.

The velocity achieved with this method (in the range of 100–500 m s^−1^) is proportional to the laser power and accordingly set by adjusting it. Solid‐state bonding of individual gold films to the gold substrate was observed above a critical impact velocity of 200 m s^−1^ (discussed in more detail in a later section). Out‐of‐plane growth to more than 20 individual voxel layers was achieved by the sequential launch of films at a constant laser power (and thus roughly constant velocity) and without translation of the receiving substrate (Figure 1e). In between each launch, a new voxel layer was centered in the focus of the ablation laser by translation of the launch pad. Currently, the available launch pad area (156 mm^2^) limits the total number of voxel layers to be transferred from a single launch pad. Hence, larger launch pads, continuous ribbon‐style launch pads, or automatic launch pad exchange need to be considered for continuous transfer of hundreds of voxel layers.

**Figure 2:**
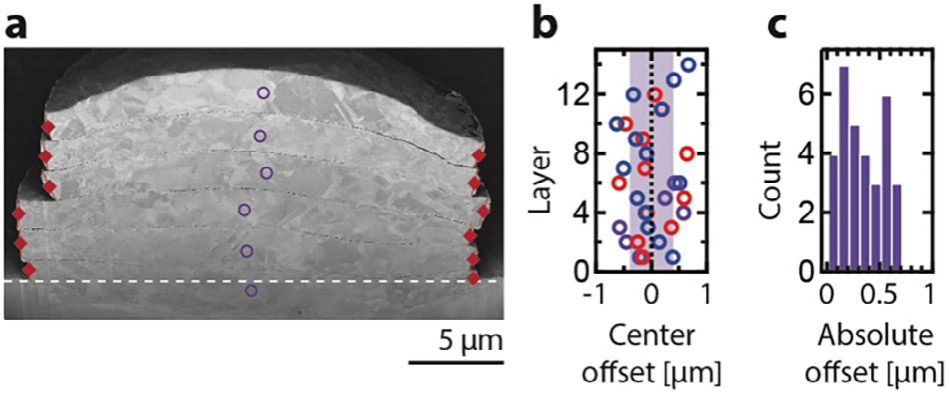
Sub‐micrometer spatial precision of transfer. a) Cross‐sectional micrograph of a stack of six layers. Purple circles mark the lateral centers of the stacks, defined as the lateral halfway position between layer edges (red diamonds). b) Offset of the center positions from the average position, as measured for three different stacks (*N* = 32). The standard deviation, ±392 nm, is shaded in purple. c) Histogram of absolute offsets from the complete dataset in (b). The average absolute offset is ±333 nm.

The spatial reproducibility of layer placement and thus the precision of the transfer is an important metric of the process. This precision was assessed by tracking the center position of individual layers (defined as the midpoint between the left and right endpoints of layers) in three cross‐sectioned stacks (23 layers in total) (**Figure** **2**). The standard deviation of the lateral position was ±392 nm, while the average lateral offset from the centerline of the stack was 333 nm (with a maximum offset of ≈700 nm). This is better than previously achieved with LIPIT of silica particles^[^ ^60^ ^]^ and comparable to 3D LIFT of congruent ink voxels.^[^ ^25^ ^]^ Currently, we believe that the main source of positional error is the manual alignment of the disks before launch, which was effected here with coarse micrometer stages and limited imaging magnification. The use of manual positioning stages also limits the launch rate to about three to six per minute. Yet, the patterned launch pad with its precisely known positions of individual elements alludes to a future automated process that addresses and fires voxel layers with even higher precision than we have attained (using alignment markers), and at a high rate (a launch rate of 10 Hz should be realistic with dynamic translation stages).

### 2.2 Variable Shape and Size of Voxel Layers

**Figure 3:**
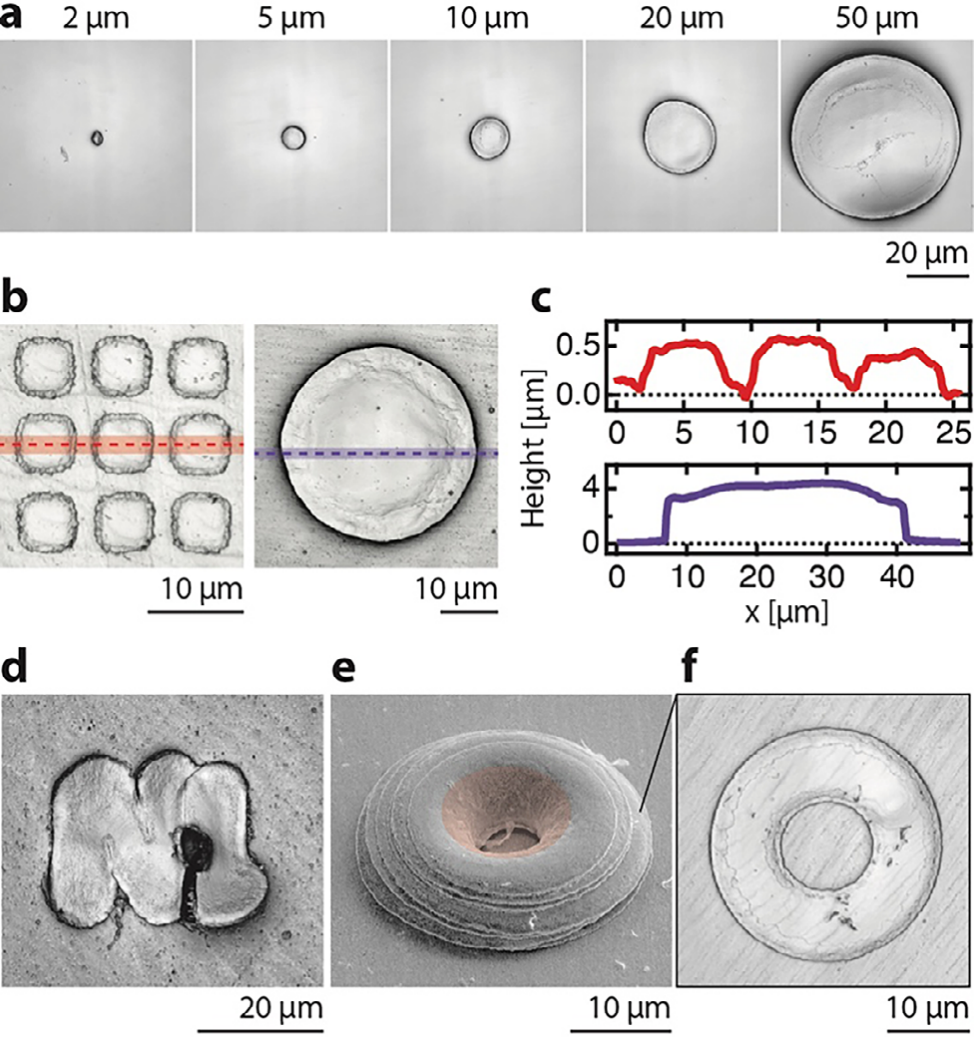
Variable layer size, thickness and shape. Laser scanning confocal micrographs of various individual, bonded gold thin‐film layers: a) Disks 2–50 µm in diameter launched in sequence from a single launch pad; b) layers of ≈0.5 and 4 µm thickness, including c) their height profiles. Shaping of voxel‐layers by lithography enables the transfer of complex shapes and the layer‐by‐layer deposition of 3D geometries. Laser‐scanning confocal micrographs of d) two bonded films shaped in the form of the letter “N” and the number “1”, transferred in parallel. e) SEM micrograph of a stack of eight rings of gradually decreasing inner and outer diameter. f) Optical micrograph of a single ring after bonding. This demonstration of layer‐by‐layer fabrication for a simple shape (dome) also indicates a challenge of fabricating overhangs: the high‐energy impact deforms unsupported parts of the layers (red shaded area). Layers in b–f) were bonded to bulk gold, whereas layers in a) were bonded to 100‐nm‐thick gold film on a silicon wafer.

To demonstrate the flexibility of the printing technique, we have bonded layers of various geometries. Voxel‐layers of variable shape, size and thickness were produced by electrodeposition of gold into resist templates produced by direct write lithography. For example, circular disks from 2 to 50 µm in diameter (**Figure** **3**a), and layers spanning thicknesses between 500 nm and 4 µm (Figure 3b) were all successfully bonded. Voxel‐layers of different shape but matched thickness were patterned onto a single launch pad, whereas layers of different thickness were launched from different launch pads (the parallel electroplating process only allows for a film of constant thickness to be plated on a single glass wafer). In general, the lower limit of voxel‐layer diameter was defined by the electroplating process. Patterns with a diameter <2 µm were not reproducibly filled during plating into the 7‐µm‐thick resist layers employed. Thinner resist films were not explored, but these should likely allow for feature sizes on the order of 500 nm when combining templated electroplating with direct write lithography. Based on the previous transfer of ≈100‐nm‐thick solid metal films by direct film ablation,^[^ ^53^, ^57^ ^]^ we assume that our indirect (and thus gentler) launch process can be applied to films well below 500 nm in thickness. The upper limit in size and thickness was defined by first, the inhomogeneous acceleration of wide shapes by the Gaussian beam profile of the ablation laser, and second, the challenge to reach the critical velocity for bonding with the increased mass of larger voxel‐layers (the acceleration of thicker, heavier films requires higher pulse energies, which can eventually fracture the front surface of the launch pad).

The volume of transferred single voxel‐layers spans more than two orders of magnitude (a factor 625 between a 2‐ and a 50‐µm wide flyer) and three orders of magnitude are within easy reach. Conceptually, a dynamic variation of the printing resolution of any AM method is desired for the best combination of minimal feature size and high throughput. Here, the nozzle‐free approach of LIFT allows for comparably rapid, precise and significant variation of the diameter of transferred elements—in principle between each launch event. Even more extreme dynamic variations of volume spanning more than three orders of magnitude were previously achieved with the laser forward transfer of congruent metal ink films using shaped beams.^[^ ^25^, ^50^ ^]^

Beyond the mere variation of layer size, a variation of their shape in principle unlocks a path toward higher throughput by facilitating the transition from voxel‐by‐voxel to layer‐by‐layer manufacturing. Figure 3c shows the bonding of two voxel‐layers shaped in the form of the letter “N” and the number “1”. These were transferred in parallel, in a single shot (the overlap is a result of excessive flattening of both layers upon impact). With the ability of transferring arbitrarily shaped 2D thin films, one can build 3D geometries from stacked layers instead of rastered voxels (Figure 3d). Here, we assembled a dome‐shaped geometry from ring‐shaped layers (e) with decreasing external and internal radii. This simple assembly of eight shaped voxel‐layers demonstrates the feasibility of layer‐by‐layer manufacturing by solid‐state transfer and bonding. At the same time though, it is apparent that the inner areas of the top layers—the areas that are unsupported by previous layers—deform upon the high‐velocity impact (red shade). One must thus conclude that the fabrication of overhanging geometries (as required for lattices and other complex 3D architectures) likely requires sacrificial support layers, in contrast to lower‐velocity transfer of viscous ink films previously used to fabricate bridging structures.^[^ ^61^ ^]^

**Figure 4:**
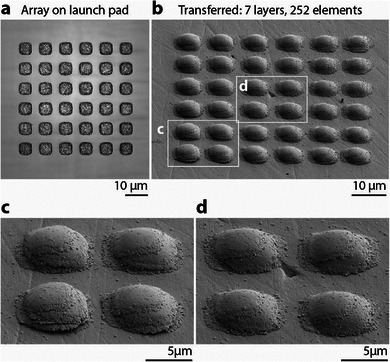
Parallel transfer. a) Laser‐scanning confocal micrograph of a 6×6 array of square gold films (≈500 nm thick, 5×5 µm, pitch 9 µm) patterned onto the launch pad. All elements in the array can be transferred simultaneously upon a single ablation shot. b–d) Array of gold stacks (seven layers in each stack, 252 elements in total) deposited in seven shots. c and d) are magnified areas of (b) (SE‐SEM micrographs, sample tilt 45°, no tilt correction).

Parallelization is yet another avenue typically explored for increased throughput. Patterning arrays of individual 5×5 µm squares, we have transferred 36 flyers in a single shot (**Figure** **4**). Stacking of layers rapidly assembles 252 such units in just seven transfer events. While this result outlines the promise of parallel transfer, it also underscores the advantage of layer thickness scaling over more than an order of magnitude demonstrated earlier. Here, stacks of seven 500 nm‐thick films could simply be replaced by one single 3.5 µm‐thick layer—a clear advantage of the transfer of pre‐shaped thick metal films by our method compared to previous results of solid‐state transfer of nm‐thick films.

### 2.3 Solid‐State Bonding

**Figure 5:**
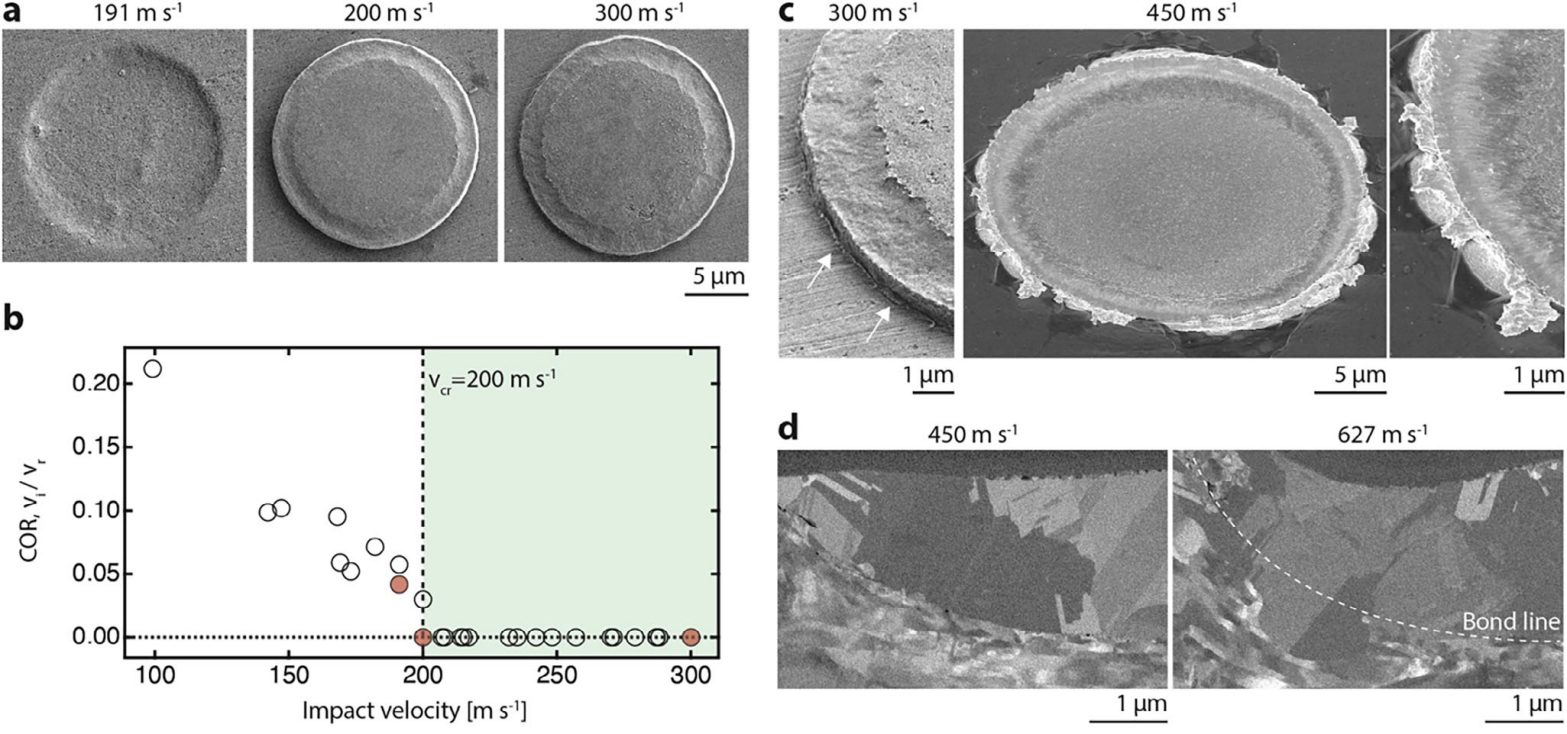
Evidence for kinetic solid‐state bonding. Bonding of single layers occurs above a critical velocity: a) SE‐SEM micrographs of impact sites after collision of gold disks with a bulk gold target at different velocities. The disks rebound from the target after impact at 191 m s^−1^ but bonded at 200 and 300 m s^−1^. b) Coefficient of restitution (COR), defined as the ratio of impact velocity to rebound velocity, v_i_/v_r_, of gold disks (14 µm diameter, 1.5 µm thick) impacting on bulk Au. Red datapoints are impacts imaged in (a). A critical velocity for bonding *v*_cr_ = 200 m s^−1^, above which all disks bond to the target, is defined from this data. c) Evidence of solid‐material jetting and extrusion is evident around the circumference of a layer impacted at 300 m s^−1^ (white arrows). After impact at 450 m s^−1^, pronounced petalling is observed (SE‐SEM, sample tilt 45°). d) Cross‐sectional micrographs of the interface between the left edge of a disk and the gold target, impacted at 450 m s^−1^ (c) and 627 m s^−1^. In the right micrograph, the growth of a recrystallized grain across the bond line evidences the formation of a flawless metallic bond (micrograph: ICD, tilt‐corrected).

A defining feature of the present approach is the bonding of cohesive metal films in their solid state. Thus, we proceed to characterize the mechanism of bond formation and the microstructural nature of the bond in more detail. A first characteristic feature of the kinetic, solid‐state bonding of metals is the existence of a critical impact velocity *v*_cr_ below which impactors rebound, and above which they bond to the impacted target.^[^ ^62^, ^63^ ^]^ Such bond formation has been related to a second characteristic feature—the onset of hydrodynamic metal jetting at the particle rims.^[^ ^63^ ^]^ Both characteristic features are observed in the present case. The post‐impact SEM micrographs in **Figure** **5**a demonstrate the rebound of a gold disk from a bulk gold target (leaving a circular indentation) upon impact at 191 m s^−1^, but their adhesion upon impact at 200 and 300 m s^−1^, respectively. As typical in microparticle impact research,^[^ ^62^, ^64^ ^]^ we quantify this behavior measuring the coefficient of restitution (COR), defined as the ratio of impact velocity to rebound velocity, *v*_i_ */v*_r_, over a wide range of impact velocities of 14 µm‐wide, 1.5 µm‐thick gold disks impacting on a bulk gold substrate (Figure 5b). The angle between impacting film and gold target was always <15°. This data lets us define a critical velocity *v_cr_* = 200 m s^−1^ above which all disks adhere to the target. This critical velocity is in good agreement with *v*_cr_ measured for spherical gold particles of similar size scale (253 m s^−1^, 16 µm diameter).^[^ ^64^ ^]^ Given that bonding velocity is typically size‐dependent in microparticles,^[^ ^65^ ^]^ we note that this critical velocity applies for 14 µm disks only. As a second characteristic, we observe pronounced extrusion of material at the edges of the disk when impacted at higher velocities (Figure 5c). While the extent of deformation is minor at 300 m s^−1^ (white arrows), pronounced petalling is observed at 450 m s^−1^. Characteristically, the ragged edges indicate severe plastic deformation that occurred in the solid state (melting would cause a smoothing of the edges^[^ ^66^ ^]^). This extrusion is characteristic of hydrodynamic jetting events that are observed in impact bonding of microparticles^[^ ^67^ ^]^ and are considered enabling of solid‐state bond formation.^[^ ^63^ ^]^ While no similar extrusion is visible in disks bonded close to the critical velocity, evidence of first jetting is found in impact craters of rebounded disks. It thus can be assumed that jetting is a phenomenon accompanying all bonding above the critical velocity, but that is sometimes obscured by the presence of the bonded layer.

Cross‐sectional microscopy further confirms the dense nature of the interface between bonded layers and suggests strong metallurgical bonding (Figure 5d). FIB‐cut cross‐sections of the interface between target and two disks impacted at 450 and 627 m s^−1^, respectively, show mostly pore‐free interfaces at the edges of the disks. At 627 m s^−1^, the layer‐target interface is partially replaced by a flawless, recrystallized grain that spans the former interface. A pronounced interfacial pore is typically found below the center of the disk, where lateral strains upon impact are nominally zero and thus little deformation of the interface is expected (as seen later, this pore is closed upon subsequent impacts). A central pore is also typically observed in impact‐bonding of spherical microparticles.^[^ ^67^ ^]^

Based on the above accumulated evidence that mirrors all features of previously described solid‐state bonding of spherical metal particles, we conclude that launched metal layers form metallurgical bonds in the solid state upon impact. Notably, this solid‐state bonding does not require a bulk metal substrate. In Figure 3a, layers were bonded to 100‐nm thin films, and this is not a lower bound on metallization layers that might serve as a base for this technique. Such films are negligibly thin in comparison to micrometer‐thick voxel‐layers and can easily be removed in a post‐transfer etching process if metal structures on insulating or semiconductive substrates are desired. Thus, the technique should be able to directly access a range of substrates.

### 2.4 High Density >99%

**Figure 6:**
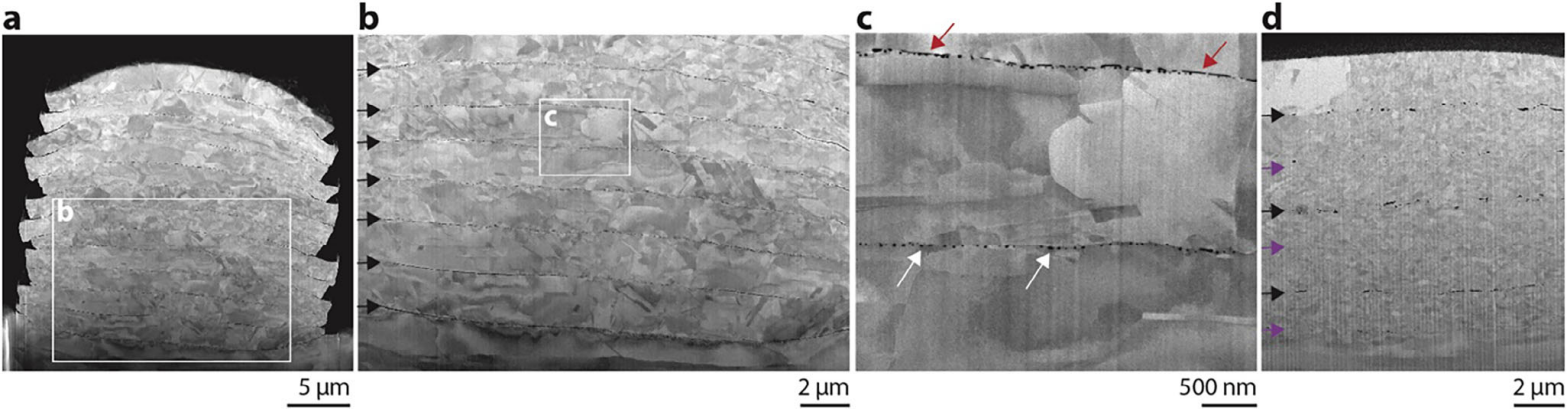
High density of stacked layers. In‐lens SEM micrographs of a–c) FIB‐milled cross‐sections of a stack of twelve layers, demonstrating the high density of deposited gold (impact velocity 200–300 m s^−1^). Pore area fraction in (b) is 0.6%. Nonetheless, interfaces between layers are clearly perceived (black arrows in b). c) Notably, the remaining porosity is a combination between incompletely bonded interfaces (red arrows) and nanopores (white arrows) already present in the electroplated layers. d) At higher impact velocities (425–500 m s^−1^), a refined microstructure and some highly densified interfaces (purple arrows) are achieved. All micrographs are tilt‐corrected.

A structure of high density develops upon stacking of multiple layers (**Figure** **6**). Because feedstock of full density is transferred, porosity is limited entirely to the interfaces between bonded layers, which are clearly seen in Figure 6b (black arrows). Notably, the interfaces are laterally homogeneous at the microscale and no evidence of significantly reduced bonding in the central region of the stack is detected (as is observed for single‐disk impacts), suggestive of a peening effect on interface development. The interface pore fraction as analyzed by image analysis of Figure 6b is only 0.6%, corresponding to a volumetric density of >99% of the complete stack, assuming the porosity is homogeneously distributed in the interfaces. It is noteworthy that the remaining porosity has two origins. First, horizontally elongated pores are clear evidence of incomplete bonding (red arrows). However, a second family of characteristic, spherical and nanoscale pores (white arrows) is already present in the sputter‐deposited seed‐layer of the electroplated films before transfer (Figure S1, Supporting Information). These pores are thus not a result of incomplete bonding. In this light, the lower interface in (c) likely is evidence of complete and flawless metallic bonding upon impact. Upon stacking at higher impact velocities (425–500 m s^−1^), extended portions of some interfaces are joined to a degree that renders them hardly detectable in the FIB‐milled cross‐section (purple arrows in (d)). These observations conform to expectations for full‐scale kinetic bonding via cold spray, which often achieves ≈99% density over many impacts in a broad range of metals.^[^ ^45^ ^]^ This high‐density microstructure obtained upon cold spray, but also upon LIPIT of multiple randomly overlapping, spherical particles,^[^ ^48^ ^]^ suggests that also in our process, rastering and overlapping of individual thin‐film voxels (instead of the stacking of layers we focused on here) can produce competitive, high densities.

### 2.5 Comparison to Alternative Metal µAM Methods

Among the metrics most appropriate to compare AM techniques are the resolution and speed, or more specifically the “minimal feature size” and “throughput”, respectively. Because of the need for high performance materials and the compatibility with CMOS technology in microfabrication, we further add “material‐focused” metrics that include “final density” and “post‐processing temperature”. The literature data that supports the quantitative comparison is listed in Table S1 (Supporting Information) and is an updated dataset from references [2] and [39].

**Figure 7:**
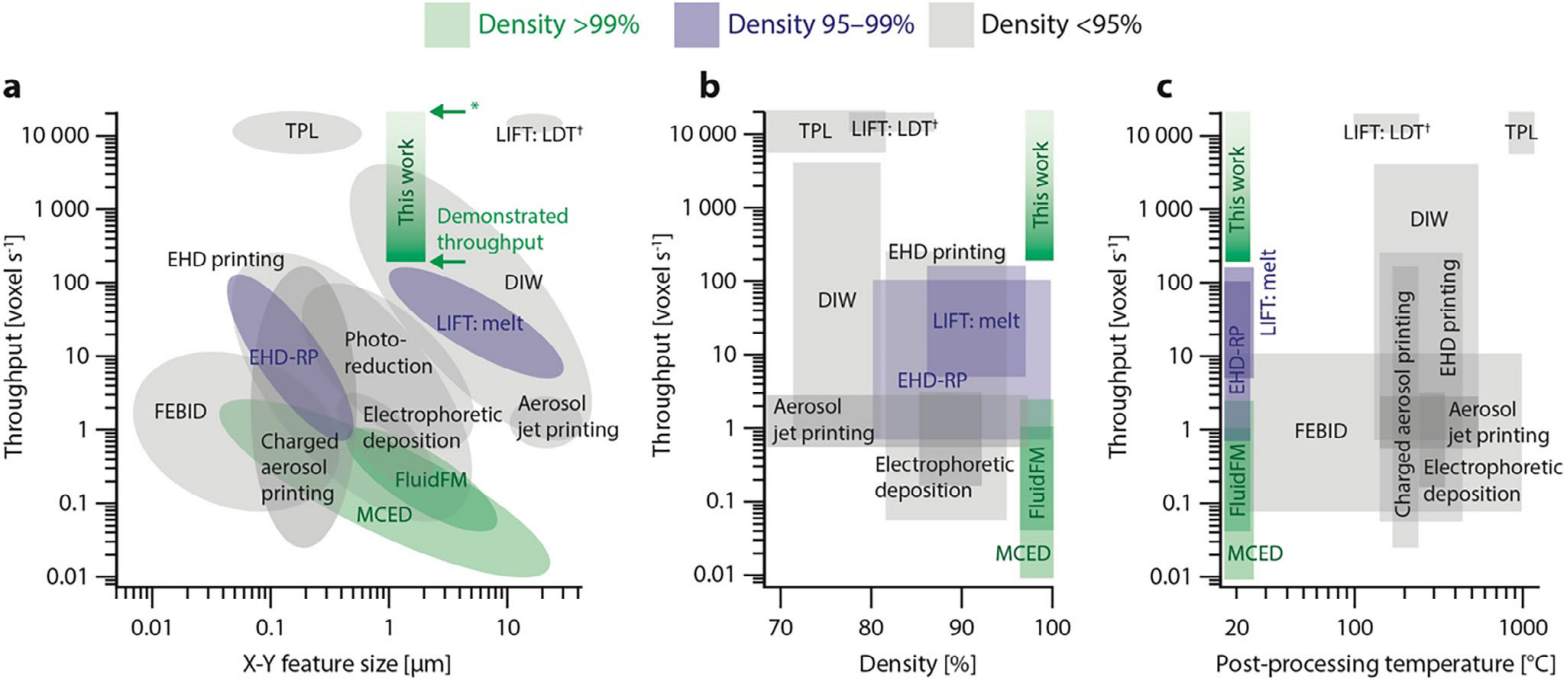
Comparison of throughput, density, feature size and processing temperature. a) A comparison of extant metal µAM methods (minimal feature size ≤10 µm) based on the parameters throughput (voxels s^−1^), lateral feature size and typically reported densities. Techniques plotted in green have reported densities of deposited metals >99%, those in yellow a typical density >95%, and those in gray <95%. (*) For our work, we display both here‐demonstrated voxel throughput (lower arrow), as well as the throughput projected for an automated system (upper arrow, 10 Hz transfer rate). (†) Similarly, the throughput of LDT is calculated with the same conservative transfer rate of 10 Hz, because the actual rate is not stated. b) A comparison of throughput and range of densities reported in literature, and c) minimal processing temperatures reported to render cohesive metals. Layer‐by‐layer kinetic solid‐state bonding occupies a unique space that combines high throughput and high density achieved without thermal annealing and the potential to rival highest throughput of all methods. Detailed data and references underlying the plots are provided in Supplementary Table S1 (Supporting Information). Abbreviations: TPL: two‐photon lithography; LDT: laser decal transfer, FEBID: focused electron beam‐induced deposition; MCED: meniscus‐confined electrodeposition. EHD‐RP: electrohydrodynamic redox printing.

An established way of comparing throughput of µAM technologies with different resolution is a normalization of volumetric throughput by voxel size (**Figure** **7**a).^[^ ^2^ ^]^ In general, the throughput of ink‐transfer methods is on the order of tens to a few thousand voxels per second, whereas aerosol‐based methods are somewhat below that range. Electrochemical methods reach up to a maximum of 100 voxels per second, and so does LIFT of metal melt droplets. Recent advances have boosted focused electron beam‐induced deposition (FEBID) up to 10 voxels per second. At a range >10 000 voxels per second, we find TPL methods as well as layer‐by‐layer manufacturing as enabled by LIFT of ink films. As a nozzle‐free technique, LIFT (or laser decal transfer, LDT)^[^ ^25^, ^50^, ^53^ ^]^ has demonstrated the ability to transfer shaped layers prefabricated on a transfer substrate and thus the potential for additive layer‐by‐layer micromanufacturing—it was unique in this capability until the present report. To compare a layer‐by‐layer based process (parallel transfer of many voxels in a single voxel‐layer) to voxel‐by‐voxel methods (sequential transfer or growth of single voxels), we define the number of voxels in a typical layer and multiply it with the transfer rate to calculate the number of voxels transferred per second. For example, we approximate the number of voxels per layer in LDT^[^ ^50^ ^]^ as follows: A grid 400 × 400 µm^2^, transferred in one shot, had a minimal feature size of 10 × 10 µm^2^. Using the latter as the lateral dimensions of a voxel, one calculates 1600 voxels per layer. A similar number is devised from the pixel‐count of the dynamic micromirror device (Table S1, Supporting Information). Because the rate of transfers per second was not given in the cited study, we resort to a conservative estimation of 10 Hz for a system that uses automated stages, and calculate a throughput of ≈15 000 voxels per second. A general comment should be made regarding the comparison of layer‐by‐layer processes to voxel‐by‐voxel methods. In our case, as well as in some LIFT of shaped ink‐films^[^ ^40^, ^68^ ^]^ or the laser transfer of prefabricated particle arrays,^[^ ^69^ ^]^ multiple fabrication steps are preformed prior to the additive transfer. These methods are thus not purely additive, and the throughput of our approach should be seen with this hybrid processing in mind. At the same time, it should be noted that these prior process steps—parallel, established and low‐cost methods for fabricating high‐quality feedstock—are neither cost‐ nor throughput‐limiting (just as the synthesis of nanoparticle inks is not a limiting factor in ink‐based methods). In fact, we propose here that such hybrid processing is a clear net benefit for increasing additive throughput and materials quality.

In our case, based on the transfer accuracy of our process and the resolution of the direct laser write lithography used to define our voxel‐layers, which are both <1 µm, we define a conservative and convenient voxel area of 1 × 1 µm^2^. In such a case, the single ring transferred in Figure 3e (≈530 µm^2^) contains roughly 500 voxels, whereas the 50 µm disk in Figure 3a (1963 µm^2^) roughly 1900 voxels. With the transfer rate of 0.1 Hz achieved with the manual translation stages, this amounts to ≈200 voxels s^−1^. At a feature size of approximately 1 µm, this throughput is comparable to that of methods like LIFT or DIW. With a conservative estimation of a 10 Hz transfer rate in a future automated system, a resulting theoretical throughput on the order of 5000–20 000 voxels/s rivals the highest numbers of any technique. This extrapolation of the present results underscores the potential of layer‐shaping and the transfer of entire voxel‐layers for high throughput.

Our process of kinetic impact‐bonding of gold thin films combines this very high throughput with an excellent density of >99% in deposited stacks. The further optimization of impact conditions, but also the surface morphology and initial porosity of voxel‐layers will be an interesting line of future work to maximize consolidation in the as‐deposited state. Yet, already now, the density achieved is significantly higher than that of metals from ink‐based AM techniques after sintering (including LIFT of ink films), is well above most other methods (including LIFT of metal melts), and is on par with electrochemical techniques^[^ ^40^ ^]^ (Figure 7b). The direct transfer of material produced from a microfabrication‐grade gold plating bath also ensures a high‐purity deposition, which is challenging for several alternative µAM methods. Notably, this performance is achieved without the need for thermal annealing or any other post‐processing. This is a significant advantage over many alternative methods, where temperatures of several hundred and up to 1000 °C for sintering or pyrolysis of organics and conversion of oxide feedstock materials into metals complicate the integration with a wide range of substrates (Figure 7c), and often is accompanied by significant volumetric shrinkage.

## 3 Conclusion

We have presented an approach based on kinetic impact‐bonding for micro additive manufacturing, which reliably deposits micrometer‐thick, solid layers into microscale 3D stacks. By lithographic patterning, we produced voxel‐layers of varying size and shape, composed of many hundreds of voxels that can be transferred in a single shot, thus enabling layer‐by‐layer (highly parallel) instead of voxel‐by‐voxel (serial) additive manufacturing. A smallest diameter of transferred features of 2 µm, and a sub‐micrometer lateral precision of stacking voxel‐layers (≈±400 nm) firmly establish the method in the realm of µAM (feature size <10 µm). In contrast to many of these technologies, including all previous demonstrations by LIFT, the impact‐bonding of solid thin‐films achieves a high stack density of >99%, rivaled only by the very best materials deposited by electrochemical methods. The method thus combines the potential for very high throughput with a highest density, achieved without significant heat‐input into the sample or substrate. The consolidation of layers at room temperature is owed to the formation of metal‐metal bonds upon co‐deformation during impact above a critical velocity of 200 m s^−1^. Notably, in this work, we reproduced all the characteristics of kinetic solid‐state bonding of metals previously established in particle‐particle impacts: a critical velocity for bonding, below which impacting films rebound and above which they bond; hydrodynamic jetting of metal in the solid state around the circumference of the impactor; and metal‐to‐metal bonding evident in cross‐sectioned samples, with some interfaces between impactor and target being locally indistinguishable from bulk material.

From these findings, we can extrapolate a number of exciting advantages of the concept by comparison to existing methods that explore the same bonding principle. From the high density of a wide range of cold‐sprayed metals, we can infer a translation of the present density of stacks into geometries built from rastered voxel layers. Analogous to cold spray^[^ ^45^, ^46^ ^]^ and previous LIPIT bonding experiments,^[^ ^58^, ^62^ ^]^ we should expect a broad materials palette for the technology, including almost all ductile metals and alloys, as well as metal‐matrix composites with hard or brittle materials, facilitated by the fact that the nozzle‐free approach is compatible with a proven pipeline of scalable and cost‐competitive, high‐purity feedstock, from commercial alloy powders to thin films. Further, the room‐temperature solid‐state joining process is famous for enabling otherwise impossible materials combinations, including that of nonweldable metals that form brittle phases upon thermal treatments. In general, the single‐step, room‐temperature voxel assembly circumvents any problematic thermal post‐processing and thus renders the approach intrinsically compatible with a wide range of substrates and devices that would be compromised by high‐temperature processing. Finally, impact‐induced deformation and peening as observed in cold spray suggest future hard metals by this µAM method, significantly strengthened by strain hardening, whereas a mild post‐deposition heat treatment should enable low defect densities if high conductivity is required. We are thus looking forward to advances along many trajectories in µAM of inorganic materials enabled by the solid‐state bonding of thin films as demonstrated here.

## 4 Experimental Section

### Microfabrication of Thin‐Film Voxel‐Layers on Launch Pads

The design rationale, fabrication, and testing of launch pads for launching patterned metal thin films have been previously described elsewhere.^[^ ^60^ ^]^ In brief, the launch pad consisted of a chromium metal layer sandwiched between two round borosilicate glass slides (25 mm in diameter, 170–250 µm thick, *VWR*). Chromium layers 90 nm in thickness were RF sputter deposited (Orion 5, *AJA*, ≤2.5 10^−5^ Torr base pressure, 3 mTorr deposition pressure, 1 Å s^−1^ deposition rate) on the first slide (the back slide). Prior to deposition, slides were sonicated in DI water and plasma cleaned (0.15 mbar oxygen, 15 min, Nano, *Thierry*), and finally plasma‐treated inside the sputter chamber (3 mTorr Ar, ≈15 W). Bare front glass slides were glued to chromium‐coated back glass slides using 20 µL of a UV‐curable adhesive (NOA 61, *Norland Products*). All slides were plasma cleaned (air, PDC‐32G, *Harrick*) before gluing. The adhesive was cured with ≈10 mW cm^−2^ (320–390 nm, measured with a UV intensity meter, ACCU‐CAL‐50, *Dymax Corporation*) to ≈3 J cm^−2^ and then aged at 50 °C for 15 h.

Gold voxel layers were fabricated by templated electrodeposition directly on the front side of the pre‐assembled launch pads. A gold seed layer (50 nm) with a chromium adhesion layer (10 nm) was sputter‐coated (Orion 5, *AJA*, ≤2.5 10^−5^ Torr base pressure, 3 mTorr deposition pressure, 1 Å s^−1^ deposition rate, RF deposition) on plasma‐cleaned launch pads. Subsequently, a positive photoresist (AZ 10XT, *MicroChemicals*) was spin coated to a thickness of ≈7 µm (500 rpm (3 s), 1000 rpm (3 s), 3000 rpm, (60 s)) and soft‐baked (110 °C, 120 s). Patterns were exposed by direct laser writing (MLA 150, *Heidelberg*, exposure mode: standard, laser: 405, dose: 425, defocus: 0) and subsequently developed (2 min 45 s, AZ435 MIF, *MicroChemicals*). Gold films were electroplated into the photoresist mask (TSG‐250 plating bath, *Transene Company*; 4.3 mA cm^−2^ peak current density, 10 ms ON, 10 ms OFF, forward only, 60 °C, stirring 300 rpm) in a two‐electrode cell (platinum‐mesh counter electrode, power supply: DUPR10‐1‐3 XR, *Dynatronix*). After plating, the resist was stripped in acetone and the wafer rinsed with isopropanol, ethanol and distilled water. Finally, the gold seed layer was typically wet‐etched (1s, TFA, *Transene Company Inc*.). Some launch pads were plasma cleaned before use (0.15 mbar oxygen, 10 min, Nano, *Thierry*).

### Laser‐Induced Transfer and Bonding

A laser‐induced particle impact tester (LIPIT) was used for the transfer of thin‐film layers and its observation. Detailed descriptions of this setup have been published elsewhere.^[^ ^70^ ^]^ To launch selected voxel‐layers toward the target, an intense laser pulse (pulsed Nd:YAG, pulse width 10 ns, *λ* = 532 nm) was focused onto the launch pad (30‐mm focal length lens, minimal focal spot size of 5 µm) to ablate the buried chromium film, launching the gold layer toward the target. The velocity of impact was controlled by adjusting the laser energy between 10–20 mJ. After each successful launch, the next voxel layer was isolated by translation of the launch pad. The launch pad was typically positioned 50–200 µm from the target substrate (bulk gold, or silicon wafers sputter coated with a 100 nm‐thick gold film and a chromium adhesion layer).

Stacks were formed by sequentially impacting and bonding layers without translation of the target in‐between shots. Reliable alignment of voxels in the center of the ablation laser beam is crucial to reproducibly overlap impacts with high precision. As described earlier, two perpendicular imaging paths for the positioning of voxels were used.^[^ ^60^ ^]^ The launch and impact of individual layers was imaged with a high‐speed camera (SIMX16, *Specialised Imaging*), with a typical frame exposure time and interframe time of 5 and 45–95 ns, respectively. Flight velocities of layers were measured from these image sequences (error ≈±5% at the relevant interframe times^[^ ^48^ ^]^). A 10 µs‐long laser pulse (*λ* = 640 nm) was used for illumination of 16 frames. The magnification of all cameras was calibrated with resolution targets before experiments.

### Analysis

Laser scanning confocal microscopy was used for measuring the dimensions of voxels and deposits (VK‐X250, *Keyence*). Profile measurements were averaged over 10 lines. A Gemini 450 SEM (*Zeiss*) was used for electron microscopy. Cross‐sections of deposits were prepared in a dual‐beam FIB (Helios 660, *FEI*) with a typical final milling current of 9.3 nA.

### Significance Statement

Microscale additive manufacturing (µAM) is an enabling concept in micro‐engineering but faces a range of technological challenges: existing methods either offer high materials performance at a noncompetitive low throughput, or low materials quality paired with high throughput. Here we for the first time demonstrate that solid‐state bonding uniquely optimizes all these interests simultaneously, and enables exceptionally low materials porosity, foregoes any challenging thermal processing steps, and offers potential for the highest throughput by replacing the serial deposition of voxels with the transfer of pre‐shaped layers. Thus, the strategy of kinetic impact‐bonding has immense potential in microscale manufacturing for applications that demand high‐performance metals deposited at competitive rates.

## Conflict of Interest

The authors declare no conflict of interest.

## Author Contributions

A.R. performed conceptualization, methodology, analysis and interpretation, investigation, writing–original draft, visualization, and funding acquisition. CAS. wrote–review and editing, performed analysis and interpretation, supervision, and funding acquisition.

## Supporting information



Supporting Information

## Data Availability

The data that support the findings of this study are available from the corresponding author upon reasonable request.
