# Supplementary material for: Microscale Metal Additive Manufacturing by Solid‐State Impact Bonding of Shaped Thin Films
Source: Small. 2025 Jul 14;21(36):2503014. doi: 10.1002/smll.202503014 (PMC12423914; doi:10.1002/smll.202503014)
Supplement: Supplementary file 1 — Supporting Information [file SMLL-21-2503014-s001.pdf]

NANO · MICRO small

Supporting Information

for *Small*, DOI 10.1002/smll.202503014

Microscale Metal Additive Manufacturing by Solid-State Impact Bonding of Shaped Thin Films

Alain Reiser and Christopher A. Schuh*

Supporting Information

Microscale metal additive manufacturing by solid-state impact bonding of shaped thin films

Alain Reiser[1,2,3]*, *Christopher A. Schuh*[1,4]

[1]Department of Materials Science and Engineering, Massachusetts Institute of Technology, Cambridge, MA 02139, USA

[2]Department of Materials Science and Engineering, KTH Royal Institute of Technology, 114 28 Stockholm, Sweden

[3]Department of Materials, ETH Zürich, 8093 Zürich, Switzerland

[4]Department of Materials Science and Engineering, Northwestern University, Evanston IL 60208, USA

*E-mail: reisera@ethz.ch

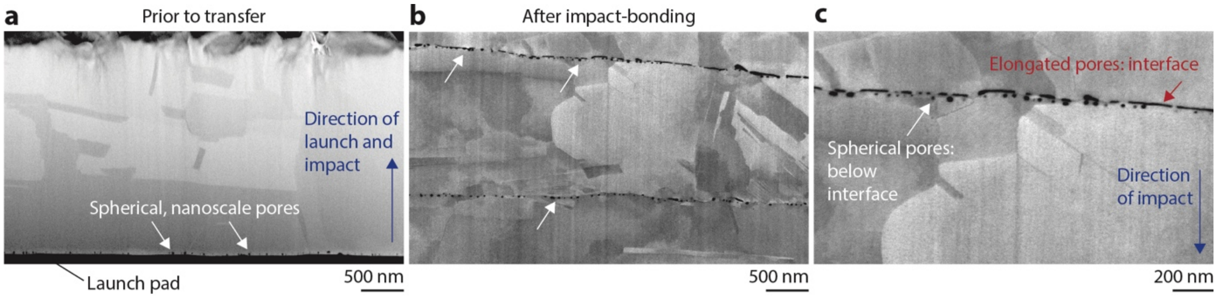

Figure S1. Interface porosity and deformation. a) In-lens SEM micrograph of the FIB-milled cross-section of a thin-film flyer prior to transfer. White arrows indicate spherical, nanoscale porosity in the sputtered gold seed layer at the interface to the glass wafer (launch pad surface). **b, c)** This characteristic porosity is transferred into stacks of flyers. Here, the porosity is located no inside the interface but clearly below the laterally elongated pores that originate from incomplete bonding and thus mark the interface between flyers. Note that the flyer in (a) is transferred upside down and collides with the top surface (transfer direction indicated by blue arrow). Thus, the spherical pores that are found above the flyer-launch pad interface in (a) are located below the flyer-flyer interface in (c). All micrographs are tilt-corrected.

Table S1. Stated densities marked with an asterisk (*) are values from image analyses performed by us (not the respective authors), using FIB cross-sections from the respective publications. Similarly, voxel rates marked with a dagger symbol (†) are our interpretation of the presented data. (–) indicates that no data was found. Much of the data that pre-dates 2017 was previously compiled in Ref [1].

Method	X-Y Feature size	Throughput	Density	Sintering temperature	Ref.
**DIW**	0.6 – 20 µm	500 – 2000 µm s^{-1}	Porous in SEM micrographs	In-situ laser annealing	[2]
	2 – 30 µm	20 – 500 µm s^{-1}	Porous in SEM micrographs	150 – 500 °C	[3]

	≈20 µm	30 – 80 µm s^{-1}	≈80% *	300 °C	[4]
**EHD printing**	0.7 – 3 µm	0.16 – 3.3 µm s^{-1}	–	150 °C	[5]
	80 – 500 nm	1 – 10 µm s^{-1} (in plane)	–	150 – 400 °C	[6]
	50 – 120 nm	Flow rate: 2 –100 µm^3 s^{-1} (0.1 vol% ink)	–	260 °C	[7]
		0.011 µm^3 s^{-1}, 11 voxel s^{-1}	96% (thin walls)	280 – 400 °C	[8]
	≈200 nm	5 µm s^{-1}	Extensive porosity in FIB cross-sections of thick structures, <89% *	400 °C	[4]
**Electrophoretic deposition**	–	0.67 µm s^{-1}	≈92% *	300 °C	[4]
	0.7 – 1.9 µm	0.37 µm s^{-1}	<90% *	Laser sintering	[9]
	0.5 – 2 µm	0.3–0.67 µm s^{-1}	–	Not performed	[10]
**LIFT: laser decal transfer (LDT)**	40 µm circular disks	–	≈80 – 87% *	150 – 230 °C	[4]
	2.5 – 500 µm	–	Porosity obvious in SEM	100 – 250 °C	[11]
	8 – 75 µm	–	–	150 – 250 °C	[12]
	Typical voxel width: 10-20 µm.	DMD: 1024 x 768 pixels. Smallest transferrable voxel: 23x23 DMD pixels (15x15 µm voxel). This amounts to 1486 voxels per voxel-layer. A 400x400 µm layer contained 1600 10x10 µm voxels.[†] No transfer rate is stated.	–	100 – 180 °C	[13]
**Aerosol jet printing**	32 – 42 µm	–	80 – 85%	350 °C	[14]
	≈10 – 20 µm	–	80 – 99%	250 – 550 °C	[15]

	≈20 – 40 µm	40 min for a lattice structure (27% fill factor) 600x600x800 µm with 35 µm-diameter trusses (=7.8x10^7 $µm^3$ or ca. 1800 voxels), ≈0.75 voxel s^{-1} †	55 – 75%	250 – 500 °C	[16]
	10 µm	2 – 5 mm/s stage translation	Porous in FIB cross section	150 – 500 °C	[17]
**Charged-aerosol jet printing**	85 – 300 nm	7–17 nm s^{-1} (300 nm pillar) ≈0.025 – 0.055 voxel s^{-1} for single pillars. With parallelization (masks with 57x57 holes): ≈80 – 180 voxel s^{-1} †	Significant porosity in FIB cross section	200 °C	[18]
**LIFT of melt droplets**	–	–	≈97% (Cu) * ≈89% (Au) *	None	[4]
	4 – 6 µm	3000 $µm^3$ s^{-1}	≈98% *	None	[19]
	7 – 40 µm	–	≈98% *	None	[20]
	6.5 µm	–	Obvious microscale porosity seen in SEM for Au	None	[21]
	5 – 7 µm	–	85 – 96%	None	[22]
**Laser-induced photoreduction**	0.18 – 1 µm	2.5 – 3 µm $s^{–1}$	Porosity obvious in SEM	None	[23]
	0.4 – 5 µm	3.4 – 50 µm $s^{–1}$	–	None	[24]
	0.5 – 3 µm	24 µm $s^{–1}$	Porosity obvious in SEM	None	[25]
**Meniscus-confined electroplating**	26 – 250 nm	Up to 70 nm/s	–	None	[26]
	12 – 15 µm	0.18 – 0.4 µm s^{-1}	–	None	[27]
	0.08–1.6 µm	0.25 – 0.35 µm s^{-1}	–	None	[28]
	0.15–0.5 µm	0.05 – 0.1 µm s^{-1}	–	None	[29]

	0.2–0.95 µm	0.25 µm s^{-1}	7970 kg m^{-3} (97% of bulk Cu)	None	[30]
	≈10 – 20 µm	0.02 – 0.4 µm s^{-1}	≈100% *	None	[4]
**FluidFM**	1 – 4 µm	Up to 40 nm/s	≈99 – 100% *	None	[31]
	0.8 – 5 µm	250 – 500 nm/s	≈99.5% *	None	[32]
	≈1 µm	10 min for a span of 33 µm	≈97 – 100% *	None	[33]
	0.4 – 0.8 µm	0.002 – 0.115 µm s^{-1}	–	None	[34]
	–	–	≈100% *	None	[4]
**EHD-RP**	85 – 250 nm	5 – 10 voxel /s	93 – 96%	None	[35]
	50 – 170 nm	10 – 100 voxel/s	80 – 99%	None	[36]
	≈150 – 500 nm	2 – 3 µm s^{-1} (out-of-plane growth rate)	"Fully dense" at optimal parameters	None	[37]
	≈100 nm (hatch distance)	5 – 40 µm s^{-1} (lateral stage translation)	≈94 – 97% *	None	[4]
	≈250 nm	4 µm s^{-1} (lateral stage translation)	Significant pores	None	[33]
**FEBID**	150 nm – 230 nm	0.2 – 0.9x10^{-3} µm^3 s^{-1}<br>0.034 – 0.026 µm s^{-1}	Nanoparticles in matrix	None	[38]
	Free rods: 8 – 20 nm<br>Tips (apex dia.): 12 – >100 nm<br>Lines (on membranes): 2–8 nm	–	Metal nanoparticles in carbonaceous matrix. An increase in purity may be achieved with thermal post-processing	Often none. But for purification and sintering: 100 – 900°C under different atmospheres	[39,40]
	15 – 50 nm	10 – 500 nm s^{-1}<br>(typical: 100 nm s^{-1})	–	–	[41]
**Two-photon litography: TPL**	50–600 nm	6700-20'000 voxel/s	70–80 %	Pyrolysis: 1000 °C<br>Reduction: 600 °C	[42]

References

[1] L. Hirt, A. Reiser, R. Spolenak, T. Zambelli, Advanced Materials 2017, 201604211, 1604211.

[2] M. A. Skylar-Scott, S. Gunasekaran, J. A. Lewis, Proceedings of the National Academy of Sciences 2016, 113, 6137.

[3] B. Y. Ahn, E. B. Duoss, M. J. Motala, X. Guo, S.-I. Park, Y. Xiong, J. Yoon, R. G. Nuzzo, J. A. Rogers, J. A. Lewis, Science (1979) 2009, 323, 1590.

[4] A. Reiser, L. Koch, K. A. Dunn, T. Matsuura, F. Iwata, O. Fogel, Z. Kotler, N. Zhou, K. Charipar, A. Piqué, P. Rohner, D. Poulikakos, S. Lee, S. K. Seol, I. Utke, C. Nisselroy, T. Zambelli, J. M. Wheeler, R. Spolenak, Adv Funct Mater 2020, 30, 1910491.

[5] B. W. An, K. Kim, H. Lee, S.-Y. Kim, Y. Shim, D.-Y. Lee, J. Y. Song, J.-U. Park, Advanced Materials 2015, 27, 4322.

[6] J. Schneider, P. Rohner, D. Thureja, M. Schmid, P. Galliker, D. Poulikakos, Adv Funct Mater 2016, 26, 833.

[7] P. Galliker, J. Schneider, H. Eghlidi, S. Kress, V. Sandoghdar, D. Poulikakos, Nat Commun 2012, 3, 890.

[8] P. Rohner, A. Reiser, F. T. Rabouw, A. S. Sologubenko, D. J. Norris, R. Spolenak, D. Poulikakos, Nanoscale 2020, 12, 20158.

[9] Y. Yoshimoto, K. Nakazawa, M. Ishikawa, A. Ono, A. Ono, F. Iwata, F. Iwata, F. Iwata, Optics Express, Vol. 31, Issue 25, pp. 41726-41739 2023, 31, 41726.

[10] T. Takai, H. Nakao, F. Iwata, Opt Express 2014, 22, 28109.

[11] E. Breckenfeld, H. Kim, R. C. Y. Auyeung, N. Charipar, P. Serra, A. Piqué, Appl Surf Sci 2015, 331, 254.

[12] J. Wang, R. C. Y. Auyeung, H. Kim, N. A. Charipar, A. Piqué, Advanced Materials 2010, 22, 4462.

[13] A. Piqué, R. C. Y. Auyeung, A. T. Smith, H. Kim, S. A. Mathews, N. A. Charipar, M. A. Kirleis, Proc. SPIE 2013, 8608, 86080K.

[14] M. S. Saleh, C. Hu, J. Brenneman, A. M. Al Mutairi, R. Panat, Addit Manuf 2021, 39, 101856.

[15] M. Sadeq Saleh, M. HamidVishkasougheh, H. Zbib, R. Panat, Scr Mater 2018, 149, 144.

[16] M. S. Saleh, C. Hu, R. Panat, Sci Adv 2017, 3, e1601986.

[17] M. S. Saleh, S. M. Ritchie, M. A. Nicholas, H. L. Gordon, C. Hu, S. Jahan, B. Yuan, R. Bezbaruah, J. W. Reddy, Z. Ahmed, M. Chamanzar, E. A. Yttri, R. P. Panat, Sci Adv 2022, 8, 4853.

[18] W. Jung, Y.-H. Jung, P. V. Pikhitsa, J. Feng, Y. Yang, M. Kim, H.-Y. Tsai, T. Tanaka, J. Shin, K.-Y. Kim, H. Choi, J. Rho, M. Choi, Nature 2021, 592, 54.

[19] C. W. Visser, R. Pohl, C. Sun, G.-W. Römer, B. Huis in ‘t Veld, D. Lohse, Advanced Materials 2015, 27, 4087.

[20] M. Zenou, A. Sa’ar, Z. Kotler, Sci Rep 2015, 5, 17265.

[21] M. Zenou, A. Sa’ar, Z. Kotler, Small 2015, 11, 4082.

[22] S. Winter, M. Zenou, Z. Kotler, J Phys D Appl Phys 2016, 49, 165310.

[23] Y. Y. Cao, N. Takeyasu, T. Tanaka, X. M. Duan, S. Kawata, Small 2009, 5, 1144.

[24] A. Ishikawa, T. Tanaka, S. Kawata, Appl Phys Lett 2006, 89, 113102.

[25] T. Tanaka, A. Ishikawa, S. Kawata, Appl Phys Lett 2006, 88, 081107.

[26] J. Hengsteler, B. Mandal, C. Van Nisselroy, G. P. S. Lau, T. Schlotter, T. Zambelli, D. Momotenko, Nano Lett 2021, 21, 9093.
[27] S. K. Seol, D. Kim, S. Lee, J. H. Kim, W. S. Chang, J. T. Kim, Small 2015, 11, 3896.
[28] J. Hu, M.-F. Yu, Science (1979) 2010, 329, 313.
[29] A. P. Suryavanshi, M.-F. Yu, Nanotechnology 2007, 18, 105305.
[30] A. P. Suryavanshi, M.-F. Yu, Appl Phys Lett 2006, 88, 083103.
[31] C. van Nisselroy, C. Shen, T. Zambelli, D. Momotenko, Addit Manuf 2022, 53, 102718.
[32] L. Hirt, S. Ihle, Z. Pan, L. Dorwling-Carter, A. Reiser, J. M. Wheeler, R. Spolenak, J. Vörös, T. Zambelli, Advanced Materials 2016, 28, 2311.
[33] M. Menétrey, C. van Nisselroy, M. Xu, J. Hengsteler, R. Spolenak, T. Zambelli, RSC Adv 2023, 13, 13575.
[34] D. Momotenko, A. Page, M. Adobes-Vidal, P. R. Unwin, ACS Nano 2016, 10, 8871.
[35] A. Reiser, M. Lindén, P. Rohner, A. Marchand, H. Galinski, A. S. Sologubenko, J. M. Wheeler, R. Zenobi, D. Poulikakos, R. Spolenak, Nat Commun 2019, 10, 1853.
[36] M. Menétrey, C. Kupferschmid, S. Gerstl, R. Spolenak, Small 2024, DOI 10.1002/smll.202402067.
[37] M. Menétrey, L. Koch, A. Sologubenko, S. Gerstl, R. Spolenak, A. Reiser, Small 2022, 2205302.
[38] H. Plank, C. Gspan, M. Dienstleder, G. Kothleitner, F. Hofer, Nanotechnology 2008, 19, 485302.
[39] I. Utke, S. Moshkalev, P. Russell, Eds., Nanofabrication Using Focused Ion and Electron Beams, Oxford University Press, Oxford, 2012.
[40] A. Botman, J. J. L. Mulders, C. W. Hagen, Nanotechnology 2009, 20, 372001.
[41] R. Winkler, J. D. Fowlkes, P. D. Rack, H. Plank, J Appl Phys 2019, 125, DOI 10.1063/1.5092372.
[42] A. Vyatskikh, S. Delalande, A. Kudo, X. Zhang, C. M. Portela, J. R. Greer, Nat Commun 2018, 9, 593.
